# Pregnancy outcomes of Chinese women undergoing IVF with embryonic cryopreservation as compared to natural conception

**DOI:** 10.1186/s12884-020-03486-7

**Published:** 2021-01-09

**Authors:** Jingxue Wang, Qiwei Liu, Boer Deng, Fang Chen, Xiaowei Liu, Jiumei Cheng

**Affiliations:** 1 Department of Minimally Invasive Gynecologic Center, Beijing Obstetrics and Gynecology Hospital, Capital Medical University, No. 17 Qihelou Road, Dong Cheng District, Beijing, China; 2 Department of Obstetrics, Beijing Obstetrics and Gynecology Hospital, Capital Medical University, No. 251 Yaojiayuan Road, Chao Yang District, Beijing, China

**Keywords:** Reasons for IVF, Infertility etiology, Adverse pregnancy outcomes, Neonatal outcomes

## Abstract

**Background:**

To examine differences in the maternal characteristics and pregnancy outcomes of Chinese women with various causes of infertility who underwent in vitro fertilization (IVF) with embryonic cryopreservation treatment.

**Methods:**

Cases were pregnancies after IVF-ET with embryonic cryopreservation; controls were spontaneously conceived pregnancies. Subgroup analysis was carried out according to etiology of infertility. The IVF treatment group was divided into 5 subgroups according to infertility etiology as follows: ovulation disorder, tubal disease, male infertility, endometriosis, and mixed infertility. Data on demographic characteristics, medical history, laboratory tests, and delivery were reviewed. Logistic regression analysis was performed for pregnancy and perinatal complications and neonatal outcomes. The multivariable model was adjusted for potential confounders.

**Results:**

Among singleton pregnancies, compared with spontaneous pregnancies, IVF pregnancies were associated with significant increases in the rates of the following: gestational diabetes mellitus (GDM) (aOR 1.76[95% CI 1.33–2.33]), preeclampsia (2.60[1.61–4.20]), preterm preeclampsia (4.52[2.03–10.06]), postpartum hemorrhage (1.57[1.04–2.36]), intrahepatic cholestasis of pregnancy (3.84[1.06–13.94]), preterm premature rupture of membranes (2.11[1.17–3.81]), preterm birth (1.95[CI 1.26–3.01]), low birthweight (1.90[1.13–3.20]), macrosomia (1.53[1.03–2.27]), and neonatal intensive care unit (NICU) admission (1.69[1.22–2.34]) in the ovulation disorder group; GDM (1.50[1.21–1.86]), placenta previa (2.70[1.59–4.59]), placenta accreta (1.78[1.10–2.89]), postpartum hemorrhage (1.61[1.19–2.18]), macrosomia (1.60[1.21–2.13]) and 5-min Apgar score ≤ 7 (4.09[1.04–16.08]) in the tubal disease group; placenta previa (9.33[4.22–20.62]), small for gestational age (2.29[1.04–5.08]), macrosomia (2.00[1.02–3.95]) and NICU admission (2.35[1.35–4.09]) in the endometriosis group; placenta previa (4.14[2.23–7.68]) and placenta accreta (2.05[1.08–3.87]) in the male infertility group; and GDM (1.85[1.15–2.98]), placenta previa (4.73[1.83–12.21]), placental abruption (3.39[1.20–9.56]), chorioamnionitis (2.93[1.04–8.26]), preterm birth (2.69[1.41–5.15]), and 1-min Apgar score ≤ 7 (4.68[1.62–13.51]) in the mixed infertility group. Among multiple pregnancies, most of the differences that were significant in singleton pregnancies were less extensive or had disappeared.

**Conclusions:**

Infertility etiology within the IVF population was found to affect maternal and neonatal outcomes among all births. During the perinatal period, infertility etiology appears to be an additional risk factor for abnormal pregnancy outcomes besides the use of IVF techniques compared with spontaneous pregnancies. Higher risk was found for ovulation disorders, and lower risk was found for male infertility.

## Background

The number of pregnancies and births after assisted reproductive technology (ART) has increased exponentially over the past 40 years. In China, ART contributes to 1% of all births [1]. Indeed, ART, such as in vitro fertilization (IVF) and intracytoplasmic sperm injection (ICSI), has become among the most important treatments for infertility. However, in recent years, evidence has emerged that ART pregnancies are at an increased risk of maternal complications and adverse pregnancy outcomes, including pregnancy-induced hypertension (PIH), gestational diabetes mellitus (GDM), placenta previa, placental abruption, postpartum hemorrhage, preterm birth, low birth weight, and small for gestational age, in both multiple pregnancies and singleton pregnancies [2–7]. In addition, some research has shown that infants conceived after ART have a higher prevalence of certain birth defects. Assisted hatching and the diagnosis of an ovulation disorder are marginally associated with increased risks for nonchromosomal birth defects [8–10]. However, other research has shown that ART is associated with a slightly elevated risk of birth defects and that the risks vary depending on the exposure [11]. However, other studies have concluded that the ART procedures associated with IVF are not responsible for adverse perinatal complications [12] because subfertile women who conceived without the aid of ART exhibited an increased risk for these adverse outcomes. Several maternal factors associated with infertility may contribute to adverse obstetric and perinatal outcomes. Despite the widespread application of ART, concerns about potential health implications remain, and the results of previous studies are controversial, partly because of their different study designs, ethnic group compositions, ART protocols and techniques used, and maternal biometric characteristics.

The reasons for the increase in adverse pregnancy outcomes with ART are unknown. It is difficult to identify whether the adverse outcomes observed with ART are the direct result of the patients’ characteristics, including type of subfertility or other factors such as cardiovascular maladaptation. One hypothesis is that an infertility-related diagnosis in a woman undergoing ART contributes directly to adverse outcomes, and excess perinatal morbidities have been associated with the infertility-related diagnosis in both ART-treated and non-ART-treated women [13]. However, after adjustments for maternal characteristics, other studies have reported few cases in which underlying infertility directly contributed to adverse outcomes [14]. Another possibility is that adverse outcomes result from the ART procedure itself, including the artificial induction of ovulation; exposure of oocytes, sperm, and embryos to the environment outside of the body; and freezing and manipulation of oocytes and embryos. In several prior studies, age-matching of patients between the ART and spontaneous conception groups was not performed. Knowledge of ART pregnancy outcomes in China is limited, and few studies have examined the relationship between infertility etiology and pregnancy outcomes.

The aim of this retrospective cohort study was to explore the impact of infertility etiology of frozen-thawed IVF on pregnancy outcomes, adjusting for maternal characteristics.

## Methods

### Data source and study sample

We conducted a large retrospective, hospital-based cohort study in couples who underwent IVF treatment at Beijing Obstetrics and Gynecology Hospital between January 2009 and May 2018. We applied the STROBE guidelines in the methods. All IVF-derived pregnancies were randomly matched to a sample of spontaneous pregnancies for maternal age and birth year. The inclusion criteria were as follows: 1) all patients were Chinese; 2) all patients in each group had live births and a gestational age of ≥28 weeks (since the perinatal period starts at 28 complete weeks in China, only gestational ages above 28 weeks are included); 3) the IVF-ET method was frozen-embryo transfer, including both blastocyst transfer and cleavage-stage embryo transfer; and 4) the infertility diagnosis was an ovulation disorder, tubal disease, endometriosis, male infertility, or mixed infertility (meaning multiple infertility-related diagnoses). The exclusion criteria were as follows: 1) the use donor oocytes/sperm or embryos, to ensure that all embryos transferred were autologous; 2) the use of preimplantation genetic testing (PGT); 3) the existence of chronic prepregnancy complications, to ensure that only patients with complications that occurred during pregnancy were studied; 4) unexplained infertility, since in our system, the diagnosis of unexplained infertility not only includes the diagnosis of infertility that did not find clear reasons but also includes the diagnosis of ovulation disorder, tubal disease, endometriosis, or male infertility, which may not be recorded clearly; if we include the unknown infertility diagnosis in our study group, bias may be introduced; 5) ICSI and other methods of conception different from IVF; or 6) women who smoked or consumed alcohol during pregnancy, to prevent confounding effects on outcomes by these factors. Overall, a total of 8773 deliveries were subjected to this retrospective analysis. Among the women, 21% (1843) had received IVF treatment. The IVF group consisted of 1241 singleton and 602 twin pregnancies. The spontaneously conceived group consisted of 6832 singleton and 98 twin pregnancies. All data, including infertility diagnosis, pregnancy, and obstetric and neonatal outcomes, were obtained from records of the patients’ visits to hospitals. The demographic and selected maternal characteristics, pregnancy and labor complications and neonatal outcomes were compared between the two groups.

### Variables of interest and definition of main outcomes

The selected maternal and pregnancy characteristics and pregnancy outcomes included the following: gestational hypertension (BP ≥140/90 mmHg after 20 weeks in previously normotensive women), preeclampsia (hypertension and proteinuria, evidence of other maternal organ dysfunction, or uteroplacental dysfunction), preterm pre-eclampsia (if preeclampsia occurred at < 37 gestational weeks) [15], GDM (diabetes diagnosed during pregnancy) [16], delivery method, intrahepatic cholestasis of pregnancy (ICP, characterized by an underlying elevation in circulating bile acids and liver derangement) [17], placenta previa (lower placenta edge within 2 cm from the internal os) [18], placenta accreta (a spectrum disorder ranging from abnormally adherent to deeply invasive placental tissue) [18], placental abruption (a premature separation of the placenta before delivery) [19], preterm premature rupture of membranes (pPROM, membrane rupture before labor and before 37 weeks of gestation) [20], chorioamnionitis (histological or clinical) [21], postpartum hemorrhage (an estimated blood loss in excess of 500 ml after a vaginal birth or a loss of greater than 1000 ml after a caesarean birth) [22], polyhydramnios (US assessment showing a largest, deepest pool of AF greater than 8 cm or an amniotic fluid index greater than 25 cm), oligohydramnios (US assessment showing a largest, deepest pool of AF less than 2 cm or an amniotic fluid index less than 2 cm) [23], preterm birth (PB or PTB, delivery after at least 28 weeks’ gestation but no more than 37 weeks’ gestation) [24], low birthweight (LBW, birthweight < 2500 g) [24], macrosomia (birth weight ≥ 4000 g) [25], small for gestational age (SGA, defined as birth weight below the 10th percentile of a standard optimal reference population for a given gestational age and sex) [26], Apgar score at 1 min, Apgar score at 5 min and neonatal intensive care unit (NICU) admission.

### Ethics approval

This study was approved by the local institutional ethics committee, namely, The Beijing Obstetrics and Gynecology Hospital committee (ethics approval number: 2019-KY-024-01), and was conducted in accordance with the Declaration of Helsinki. Due to the retrospective study design, consent for participation was not required. Nevertheless, private information was well-protected during the study.

### Statistical analysis

SPSS statistical software (version 20.0) was used for data analysis. We first compared baseline characteristics between all IVF groups vs natural pregnancies. Quantitative data are presented as the mean and SD (mean ± SD). Fisher’s exact tests, *t* tests and Pearson’s chi-square tests were performed to evaluate differences in the proportions of categorical variables between two or more groups. Second, we assessed the effect of infertility diagnosis on adverse perinatal and neonatal outcomes by comparing the prevalence rates of adverse perinatal and neonatal outcomes in different infertility diagnosis subgroups and natural pregnancies. Logistic regression analysis was conducted to calculate approximate relative risks of adverse outcomes and to identify possible predictors of pregnancy complications. The multivariable model was adjusted for maternal age, gravidity, parity, prepregnancy obesity (body mass index≥28 kg/m^2^) [27], birth plurality, and history of previous caesarean section; the results are reported as adjusted odds ratios (aORs) and 95% confidence intervals (CIs). Centiles birthweight were calculated from reference equation of published dataset on similar population and study groups [28, 29]. *P* values of less than 0.05 were considered statistically significant. The methods were carried out in accordance with approved guidelines.

## Results

**Fig. 1:**
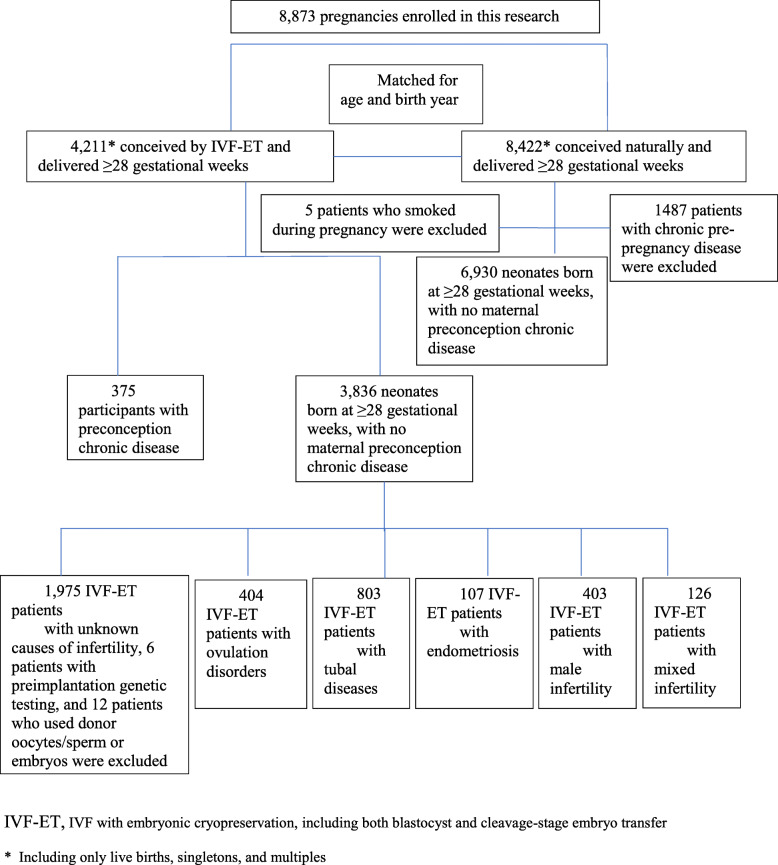
Flow chart of participants in the analysis

Figure 1 shows the flow chart of the participants who were either included in the main analysis or excluded for failing to meet the inclusion criteria. The diagnosis for IVF-treated deliveries included ovulation disorders (*N* = 404), tubal disease (*N* = 803), endometriosis (*N* = 107), male infertility (*N* = 403), and mixed infertility (*N* = 126). The number of natural pregnancies was 6930. Table 1 summarizes the background characteristics of the women who were included in the main analysis. Women with IVF pregnancies were more likely to have significantly higher rates of prepregnancy obesity, caesarean section, and multiple pregnancy and a lower rate of previous caesarean delivery than women with spontaneous pregnancies (*P < 0.001*). The spontaneous pregnancy group also had a significantly higher number of second gravidity and pregnancies than the IVF group (*P < 0.001*).

**Table 1:** Maternal and pregnancy characteristics of the ART and spontaneous pregnancy groups

	Ovulation disorder Ovulation disorder disorder	Tubal disease	Endometriosis	Male infertility	Mixed infertility	All ART	Controls	*P* value*
Number of patients	404	803	107	403	126	1843	6930	
Maternal age	33.6±4.15	33.33±3.10	34.11±3.58	33.41±3.50	33.27±3.50	33.40±3.50	33.39±2.96	0.11
Gravidity								<0.001
G1	258 (63.9%)	436 (54.3%)	74 (69.2%)	289 (71.7%)	75 (59.5%)	1132 (61.4%)	2879 (41.6%)	
G ≥ 2	146 (36.1%)	367 (45.7%)	33 (30.8%)	114 (28.3%)	51 (40.5%)	711 (38.5%)	4048 (58.4%)	
Parity								<0.001
P1	382 (94.6%)	762 (94.9%)	104 (97.2%)	389 (96.5%)	124 (98.4%)	1761 (95.6%)	5055 (73%)	
P ≥ 2	22 (5.4%)	41 (5.1%)	3 (2.8%)	14 (3.5%)	2 (1.6%)	82 (4.4%)	1872 (27%)	
Pre-pregnancy obesity								<0.001
Yes	15 (3.7%)	13 (1.6%)	1 (0.9%)	7 (1.7%)	4 (3.2%)	40 (2.2%)	61 (0.9%)	
No	389 (96.3%)	790 (98.4%)	106 (99.1%)	396 (98.3%)	122 (96.8%)	1803 (97.8%)	6869 (99.1%)	
Birth plurality								<0.001
Singleton	271 (67.1%)	542 (67.5%)	74 (69.2%)	267 (66.3%)	87 (69%)	1241 (67.3%)	6832 (98.6%)	
Multiple	133 (32.9%)	261 (32.5%)	33 (30.8%)	136 (33.7%)	39 (31%)	602 (32.7%)	98 (1.4%)	
Previous caesarean delivery								<0.001
Yes	6 (1.5%)	17 (2.1%)	3 (2.8%)	6 (1.5%)	2 (1.6%)	34 (1.8%)	772 (11.1%)	
No	398 (98.5%)	786 (97.9%)	104 (97.2%)	397 (98.5%)	124 (98.4%)	1809 (98.2%)	6158 (88.9%)	
Delivery method								<0.001
Vaginal delivery	115 (28.5%)	227 (28.3%)	24 (22.4%)	115 (28.5%)	27 (21.4%)	508 (27.6%)	3547 (51.2%)	
Caesarean section	261 (64.6%)	538 (67%)	75 (70.1%)	276 (68.5%)	91 (72.2%)	1241 (67.3%)	3100 (44.7%)	
Operative vaginal delivery	28 (6.9%)	38 (4.7%)	8 (7.5%)	12 (3%)	8 (6.3%)	94 (5.1%)	283 (4.1%)	
Number of embryos transferred								
1	274 (67.2%)	545 (67.0%)	74 (67.9%)	271(66.1%)	89 (68.5%)			
≥ 2	134 (32.8%)	268 (33%)	35 (32.1%)	139(33.9%)	41 (31.5%)			

Note: Data are presented as means±SDs for continuous variables and n (%) for dichotomous variables

Mixed infertility refers to a multiple infertility-related diagnosis. Both blastocyst transfer and cleavage stage embryos transfer are included

*P values were assessed by using the Pearson’s chi-square test, Fisher’s exact test or the t test between all IVF group vs controls ; Obesity means BMI≥28 kg/m2

Table 2 shows the pregnancy outcomes among different infertility etiologies for IVF vs spontaneous pregnancies in singleton pregnancies. Table 3 shows the neonatal outcomes among different infertility etiologies for IVF vs spontaneous pregnancies in singleton pregnancies. The associations between different infertility etiologies and maternal/perinatal complication or adverse outcomes were assessed using a logistic regression, with women who conceived spontaneously serving as a reference (Table 4). In the same way, the associations between different infertility etiologies and neonatal complications or adverse outcomes were assessed using a logistic regression, with women who conceived spontaneously serving as a reference (Table 5). Among singleton pregnancies, women with ovulation disorders who conceived by IVF had higher risks of preeclampsia (aOR 2.60 [95% CI 1.61–4.20]), preterm preeclampsia (aOR 4.52 [95% CI 2.03–10.06]), GDM (aOR 1.76 [95% CI 1.33–2.33]), ICP (aOR 3.84 [95% CI 1.06–13.94]), pPROM (aOR 2.11 [95% CI 1.17–3.81]), postpartum hemorrhage (aOR 1.57 [95% CI 1.04–2.36]), PB (aOR 1.95 [95% CI 1.26–3.01]), low birthweight (aOR 1.90 [95% CI 1.13–3.20]), macrosomia (aOR 1.53 [95% CI 1.03–2.27]), and NICU admission (aOR 1.69 [95% CI 1.22–2.34]) than those who conceived spontaneously. Additionally, women with tubal disease who conceived by IVF had a higher risk of GDM (aOR 1.50 [95% CI 1.21–1.86]), placenta previa (aOR 2.70 [95% CI 1.59–4.59]), placenta accreta (aOR 1.78 [95% CI 1.10–2.89]), postpartum hemorrhage (aOR 1.61 [95% CI 1.19–2.18]), macrosomia (aOR 1.60 [95% CI 1.21–2.13]), and a 5-min Apgar score ≤ 7 (aOR 4.09 [95% CI 1.04–16.08]) than those who conceived spontaneously. Women with endometriosis who conceived by IVF had a higher risk of placenta previa (aOR 9.33 [95% CI 4.22–20.62]), SGA (aOR 2.29 [95% CI 1.04–5.08]), macrosomia (aOR 2.00 [95% CI 1.02–3.95]), and NICU admission (aOR 2.35 [95% CI 1.35–4.09]) than those who conceived spontaneously. Women with male infertility who conceived by IVF had a higher risk of placenta previa (aOR 4.14 [95% CI 2.23–7.68]) and placenta accreta (aOR 2.05 [95% CI 1.08–3.87]). Women with mixed infertility who conceived by IVF had a higher risk of GDM (aOR 1.85 [95% CI 1.15–2.98]), placenta previa (aOR 4.73 [95% CI 1.83–12.21]), placental abruption (aOR 3.39 [95% CI 1.20–9.56]), chorioamnionitis (aOR 2.93 [95% CI 1.04–8.26]), PB (aOR 2.69 [95% CI 1.41–5.15]), and a 1-min Apgar score ≤ 7 (aOR 4.68 [95% CI 1.62–13.51]) than those who conceived spontaneously. However, when twin pregnancies were compared with spontaneous pregnancies, only the rates of the following were significantly increased: GDM in the ovulation disorder and mixed infertility groups and 1-min Apgar score ≤ 7 in the mixed infertility group; the other differences that were significantly higher in the singleton pregnancy cohort had narrowed or disappeared in the twin pregnancy cohort (see Supplementary Tables 1–4).

**Table 2:** Pregnancy outcomes among different infertility etiologies for IVF compared to spontaneous pregnancies in singleton pregnancies

	Control		Ovulation disorder		Tubal disease		Endometriosis		Male infertility		Mixed infertility	
Singleton pregnancies	N	%	N	%	N	%	N	%	N	%	N	%
Maternal outcomes	6832		271		542		74		267		87	
Gestational hypertension	271	4	13	4.8	18	3.3	3	4.1	18	6.7^	2	2.3
Preeclampsia	193	2.8	22	8.1*	23	4.2	2	2.7	9	3.4	4	4.6
Mild	63	0.9	8	3*	7	1.3	0	0	2	0.2	1	1.1
Severe	130	1.9	14	5.2*	16	3	2	2.7	7	2.6	3	3.4
Preterm preeclampsia	46	0.7	8	3*	5	0.9	0	0	3	1.1	2	2.3
GDM	1132	16.6	76	28*	129	23.8*	15	20.3	57	21.3^	25	28.7^
Placenta previa	94	1.4	3	1.1	18	3.3^	8	10.8^	13	4.9*	5	5.7*
Placental abruption	92	1.3	7	2.6	7	1.3	2	2.7	2	0.7	4	4.6^
pPROM	154	2.3	13	4.8^	19	3.5	3	4.1	11	4.1	3	3.4
Placenta accreta	148	2.2	7	2.6	20	3.7^	3	4.1	11	4.1^	3	3.4
Postpartum haemorrhage	574	8.4	31	11.4	59	10.9^	6	8.1	21	7.9	9	10.3
ICP	16	0.2	3	1.1^	4	0.7^	0	0	1	0.4	0	0
Polyhydramnios	61	0.9	4	1.5	7	1.3	1	1.4	5	1.9	0	0
Oligohydramnios	247	3.6	14	5.2	18	3.3	4	5.4	12	4.5	2	2.3
Chorioamnionitis	91	1.4	7	2.6	10	1.8	2	2.7	3	1.1	4	4.6^

Note: *GDM* Gestational diabetes mellitus, *pPROM* preterm premature rupture of membranes, *ICP* intrahepatic cholestasis of pregnancy; Mixed infertility refers to multiple infertility-related diagnosis. Data are presented as n (%) for dichotomous variable, *P* values were assessed by using the Pearson’s chi-square test, or Fisher’s exact test between each infertility subgroup vs controls ; *P* ≤ 0.001 = *; *P* < 0.05 = ^. Nonsignificant numbers have not symbol

**Table 3:** Neonatal outcomes among different infertility etiologies for IVF compared to spontaneous pregnancies in singleton pregnancies

	Control		Ovulation disorder		Tubal disease		Endometriosis		Male infertility		Mixed infertility	
Singleton Pregnancies	N	%	N	%	N	%	N	%	N	%	N	%
	6832		271		542		74		267		87	
Gestational weeks	38.8±1.5		38.3±1.8*		38.5±1.6*		38.3±1.8^		38.5±1.8^		38.1±1.9*	
Preterm birth	355	5.2	25	9.2^	36	6.6	7	9.5	20	7.5	11	12.6^
34≤PTD<37 wk	267	3.9	17	6.3	29	5.4	5	6.8	16	6	9	10.3^
28≤PTD<34 wk	88	1.3	8	3^	7	1.3	2	2.7	5	1.9	2	2.3
Birthweight, g( Mean±SD)	3370±474		3379±580		3382±498		3244±502^		3335±519		3278±518	
Birthweight centile(Mean±SD)	57.29±24.81		59.51±26.60		57.29±26.02		51.84±27.97		55.28±26.80		57.37±26.53	
< 2500 g	236	3.5	17	6.3^	21	3.9	4	5.4	13	4.9	6	6.9
< 1500 g	38	0.6	4	1.5	3	0.6	0	0	3	1.1	1	1.1
SGA	281	4.1	18	6.6^	21	3.9	7	9.5^	12	4.5	5	5.7
Macrosomia	521	7.6	31	11.4^	64	11.8*	10	13.5	20	7.5	6	6.9
Body length, cm	50.55±41.78		49.85±2.37		50.02±1.88		49.73±2.01		49.92±2.04		49.63±2.12^	
1-Minute Apgar score≤7	64	0.9	5	1.8	4	0.7	0	0	4	1.5	4	4.6*
5-Minute Apgar score≤7	10	0.1	1	0.4	3	0.6*	0	0	0	0	0	0
NICU admission	728	10.7	48	17.7*	72	13.3	17	23*	33	12.4	15	17.2^

Note: *PTD* preterm delivery, *SGA* small for gestational age( birthweight below the 10th percentile for gestational age); Macrosomia = birth weight ≥ 4000g

Mixed infertility refers to multiple infertility-related diagnosis

Data are presented as means±SDs for continuous variables and n (%) for dichotomous variables. *P* values were assessed by using the Pearson’s chi-square test, Fisher’s exact test or the *t* test between each infertility subgroup vs controls ; *P* ≤ 0.001 = *; *P* < 0.05 = ^. Nonsignificant numbers have not symbol

**Table 4:** cORs, aORs and 95% CIs of pregnancy and delivery outcomes among different infertility etiologies compared to spontaneous pregnancies in singleton pregnancies

	Ovulation disorder				Tubal disease				Endometriosis				Male infertility				Mixed infertility			
SP	cOR	aOR	95% CI		cOR	aOR	95% CI		cOR	aOR	95% CI		cOR	aOR	95% CI		cOR	aOR	95% CI	
GH	**0.63**	0.93	0.52	1.68	0.91	0.72	0.44	1.19	1.06	0.90	0.28	2.89	**0.55**	1.49	0.90	2.47	1.76	0.45	0.11	1.85
Preeclampsia	**0.26**	**2.60**	**1.61**	**4.20**	**0.44**	1.40	0.89	2.21	0.75	0.90	0.22	3.74	**0.44**	1.10	0.55	2.20	0.60	1.39	0.50	3.90
Mild	**0.30**	**2.67**	**1.23**	**5.79**	0.57	1.20	0.54	2.68	0.99	NC	NC	NC	0.93	0.67	0.16	2.77	0.80	0.98	0.13	7.19
Severe	**0.25**	**2.46**	**1.36**	**4.44**	**0.40**	1.51	0.88	2.59	0.67	1.42	0.34	5.92	**0.35**	1.36	0.62	2.99	0.54	1.62	0.49	5.29
**PPE**	**0.10**	**4.52**	**2.03**	**10.06**	**0.22**	1.38	0.54	3.54	0.72	NC	NC	NC	**0.18**	1.66	0.50	5.49	0.29	3.37	0.79	14.46
GDM	**0.43**	**1.76**	**1.33**	**2.33**	**0.72**	**1.50**	**1.21**	**1.86**	0.73	1.20	0.67	2.13	**0.72**	1.31	0.96	1.77	**0.49**	**1.85**	**1.15**	**2.98**
Placenta previa	1.40	0.93	0.29	3.00	**0.47**	**2.70**	**1.59**	**4.59**	**0.14**	**9.33**	**4.22**	**20.62**	**0.39**	**4.14**	**2.23**	**7.68**	**0.23**	**4.73**	**1.83**	**12.21**
Placental abruption	0.68	1.89	0.86	4.17	1.36	0.92	0.42	2.01	0.72	1.90	0.46	7.97	1.82	0.55	0.13	2.25	**0.28**	**3.39**	**1.20**	**9.56**
pPROM	**0.23**	**2.11**	**1.17**	**3.81**	**0.36**	1.52	0.93	2.49	0.47	1.76	0.55	5.70	**0.27**	1.79	0.95	3.37	0.65	1.49	0.46	4.80
Placenta accreta	NC	1.29	0.59	2.80	**NC**	**1.78**	**1.10**	**2.89**	NC	2.00	0.62	6.48	**NC**	**2.05**	**1.08**	**3.87**	NC	1.70	0.53	5.48
PH	**0.65**	**1.57**	**1.04**	**2.36**	**0.65**	**1.61**	**1.19**	**2.18**	0.66	1.11	0.46	2.68	0.73	1.17	0.73	1.88	0.80	1.61	0.77	3.36
ICP	**0.13**	**3.84**	**1.06**	**13.94**	**0.16**	2.77	0.89	8.60	NC	NC	NC	NC	**0.19**	1.36	0.17	10.58	NC	NC	NC	NC
Polyhydramnios	0.51	1.72	0.60	4.89	0.80	1.56	0.70	3.48	0.96	1.79	0.24	13.24	**0.45**	2.27	0.88	5.82	NC	NC	NC	NC
Oligohydramnios	0.91	1.19	0.68	2.10	1.55	0.75	0.46	1.22	0.97	1.17	0.42	3.25	1.13	1.01	0.55	1.83	1.59	0.48	0.12	1.96
Chorioamnionitis	0.53	1.64	0.74	3.63	0.97	1.19	0.61	2.32	0.71	1.76	0.42	7.38	1.80	0.75	0.23	2.40	**0.28**	**2.93**	**1.04**	**8.26**

*CI* confidence interval, *cOR* crude odds ratio, *aOR* djusted odds ratio. Mixed infertility refers to multiple infertility-related diagnosis; *SP* singleton pregnancies, *GH* gestational hypertension, *PPE* preterm preeclampsia, *GDM* gestational diabetes mellitus, *pPROM* preterm premature rupture of membranes, *PH* postpartum haemorrhage, *ICP* intrahepatic cholestasis of pregnancy

Bold indicates significant differences; *NC* not calculated due to low numbers

Note: Logistic regression analysis was adjusted for age, gravidity, parity, pre-pregnancy obesity, birth plurality, and history of previous caesarean section. The reference group of logistic regression is spontaneous control pregnancy

**Table 5:** cORs, aORs and 95% CIs of neonatal outcomes among different infertility etiologies compared to spontaneous pregnancies in singleton pregnancies

	Ovulation disorder				Tubal disease				Endometriosis				Male infertility				Mixed infertility			
SP	cOR	aOR	95% CI		cOR	aOR	95% CI		cOR	aOR	95% CI		cOR	aOR	95% CI		cOR	aOR	95% CI	
Preterm birth	**0.18**	**1.95**	**1.26**	**3.01**	**0.27**	1.33	0.93	1.91	**0.27**	1.97	0.89	4.34	**0.21**	1.52	0.95	2.44	**0.20**	**2.69**	**1.41**	**5.15**
34≤PTD<37 wk	**0.19**	**1.73**	**1.03**	**2.89**	**0.27**	1.42	0.95	2.11	**0.27**	1.83	0.73	4.61	**0.22**	1.61	0.95	2.73	**0.20**	**2.88**	**1.42**	**5.84**
28≤PTD<34 wk	**0.21**	**2.49**	**1.17**	**5.29**	**0.38**	1.06	0.48	2.31	**0.34**	2.24	0.53	9.38	**0.24**	1.52	0.60	3.83	**0.26**	1.88	0.45	7.84
Birthweight <2500 g	**0.12**	**1.90**	**1.13**	**3.20**	**0.14**	1.12	0.71	1.78	**0.14**	1.59	0.57	4.42	**0.12**	1.42	0.80	2.54	**0.13**	2.04	0.88	4.76
Birthweight <1500 g	**0.18**	**3.31**	**1.34**	**9.60**	**0.28**	1.09	0.33	3.59	0.29	NC	NC	NC	**0.18**	2.29	0.69	7.64	**0.17**	2.38	0.32	17.81
SGA	**0.28**	1.60	0.97	2.65	**0.30**	0.90	0.57	1.42	**0.23**	**2.29**	**1.04**	**5.08**	**0.33**	1.03	0.57	1.88	**0.24**	1.36	0.54	3.40
Macrosomia	0.99	**1.53**	**1.03**	**2.27**	0.95	**1.60**	**1.21**	**2.13**	0.80	**2.00**	**1.02**	**3.95**	**1.58**	0.97	0.60	1.55	1.65	0.86	0.37	2.00
1-Minute Apgar score≤7	**0.37**	1.84	0.72	4.73	0.94	0.77	0.28	2.15	0.50	NC	NC	NC	**0.37**	1.70	0.61	4.80	0.19	**4.68**	**1.62**	**13.51**
5-Minute Apgar score≤7	**0.20**	2.39	0.27	20.34	**0.29**	**4.09**	**1.04**	**16.08**	NC	NC	NC	NC	**0.20**	NC	NC	NC	0.18	NC	NC	NC
NICU admission	**0.26**	**1.69**	**1.22**	**2.34**	**0.34**	1.24	0.95	1.61	**0.28**	**2.35**	**1.35**	**4.09**	**0.31**	1.17	0.80	1.70	**0.31**	1.61	0.92	2.84

^a^Logistic regression analysis was adjusted for age, gravidity, parity, pre-pregnancy obesity, birth plurality, and history of previous caesarean section. *CI* confidence interval, *cOR* crude odds ratio, *aOR* adjusted odds ratio

Mixed infertility refers to multiple infertility-related diagnosis, *SP* singleton pregnancies, *PTD* preterm delivery, *SGA* small for gestational age( birthweight below the 10th percentile for gestational age), *NICU* neonatal intensive care unit; Macrosomia = birth weight≥4000g; The reference group of logistic regression is spontaneous control pregnancy; Bold indicates significant differences; *NC* not calculated due to low numbers

## Discussion

### Summary of key findings

Through this retrospective, hospital-based cohort study of pregnant Chinese women, we verified that infertility etiologies within the IVF population were found to affect maternal and neonatal outcomes among all births. Among singleton pregnancies, compared with spontaneous pregnancies, IVF pregnancies with ovulation disorders have higher risks of adverse pregnancy and perinatal outcomes. The rates of the following were significantly increased: GDM (1.8-fold), preeclampsia (4.5-fold), postpartum hemorrhage (1.6-fold), pPROM (2.1-fold), and preterm birth (2-fold), while IVF pregnancies with male infertility have lower risks of adverse pregnancy and perinatal outcomes.

### Interpretation

Our study shows that ovulation disorders were associated with higher risks of many pregnancy and neonatal complications. The results are consistent with prior studies [13, 30, 31]. As the use of IVF increases and newer technologies continue to push the boundaries of science, it is important to consider the clinical safety of these approaches. One possible explanation is that a high proportion of women with ovulation disorders have polycystic ovarian syndrome (PCOS), and many of them have multiple metabolic abnormalities. Growing evidence demonstrates that PCOS has a negative impact on fertility and pregnancy outcomes, such as GDM, gestational hypertensive disorders, and PB [32]. GDM is evidently related to the delivery of an infant with macrosomia, so the incidence of macrosomia is significantly higher for pregnant women with PCOS [31]. In addition, neonates of women with PCOS are at greater risk of neonatal complications, including perinatal mortality, prematurity, SGA, lower birth weight and higher NICU admission [33]. Current evidence also suggests that prepregnancy hormonal dysfunction, including hyperandrogenism, progesterone resistance and hyperinsulinism, impairs uterine placentation mechanisms, which may lead to a greater risk of adverse obstetric and neonatal outcomes [33].

In our study, compared with spontaneous pregnancies, IVF pregnancies in patients who had tubal infertility had increased risks of GDM, placenta previa, placenta accreta, postpartum hemorrhage, macrosomia, and a 5-min Apgar score ≤ 7. One study reported that infertility, particularly due to an ovulatory disorder or tubal blockage, was associated with an increased GDM risk; specifically, women with a history of infertility due to tubal blockage had an 83% greater risk [34], consistent with our results. GDM is closely related to the birth of an infant with macrosomia; therefore, the rate of macrosomia in tubal infertility is also significantly increased. Tubal-factor infertility is always associated with reproductive inflammation, which may lead to an imbalance in immune-endocrine crosstalk among the endometrium, myometrium and cervix and between the decidua and trophoblasts, predisposing patients to pregnancy complications, such as placenta previa, placenta accreta and postpartum hemorrhage, which could affect neonatal outcomes.

Our data showed that endometriosis was significantly associated with placenta previa, SGA, and NICU admission, similar to the findings of previous studies [35–38]. Endometriosis is a common reason for infertility and may cause chronic inflammation and adhesions in the pelvis of reproductive-aged women. Moreover, women with endometriosis exhibit defective deep placentation because of defective remodeling of the spiral arteries [39]. P Cavoretto found that in a longitudinal cohort study of uterine artery Doppler in singleton pregnancies, different uterine arteries perfusion (with lower pulsatility index) occurring in frozen as compared to fresh embryo transfer pregnancy from the first to the third trimester, related to higher birth weight in frozen as compared to fresh IVF/ICSI [28]. Which may indicate that pregnancy outcomes are affected by uterine arteries prefusion. These factors may explain why endometriosis is possibly a crucial factor for increased negative outcomes in IVF pregnancy. However, Benaglia L found that women with endometriosis who conceived via IVF do not face an increased risk of preterm birth [40], similar to our findings. In addition, we found that IVF pregnancies in patients with endometriosis had a higher rate of macrosomia (2-fold) than those who conceived naturally. Regrettably, we have not found any literature on the relationship between endometriosis and macrosomia. This controversial result still needs to be further studied by expanding the sample size.

In the male infertility subgroup, the rates of placenta previa and placenta accreta were also increased, but this has not been universally reported. One possible explanation is that the increased risks of placenta previa and placenta accreta are caused by factors related to IVF [41, 42]. Indeed, the intrauterine operation and manipulation of embryonic cells in IVF might induce uterine contraction, leading to higher frequencies of implantation in the lower uterine segment, which may increase the risk of placenta previa. The changes to the endometrium wrought by IVF treatment protocols, and the use of hormone therapy to promote embryo implantation, may increase the risk of placenta accreta. In this research, the risk of placenta previa increased in all subgroups except for the ovarian disorder subgroup, which was similar to previous research [41]. Interestingly, there were no significant differences in neonatal outcomes between IVF and spontaneous conception in the male infertility subgroup. Vannuccini S found that in uncomplicated term pregnancies following ART, infants born after ART had similar birthweights, Apgar scores and arterial blood pH values as those of spontaneously conceived infants [43]. These findings might indicate that the factors associated with infertility are more likely to be associated with adverse neonatal complications rather than the ART procedure itself, which is consistent with a previous study [44]. Overall, the results require further analysis in larger cohorts, adjustments for as many confounders as possible and further preclinical studies.

Recent evidence with meta-analysis show the impact of IVF/ICSI pregnancy which more than doubles the rate of iatrogenic preterm birth [45]. In our study, indirect subanalysis, on cryopreservation fail to achieve statistical significance for iatrogenic preterm birth, however use of cryopreservation present possible association with reduction of the rate of spontaneous preterm birth.

Our study also showed increased risks for GDM, placenta previa, chorioamnionitis, PB, and a 1-min Apgar score ≤ 7 in the mixed infertility subgroup compared with corresponding controls. When there are mixed reasons for parental infertility, pregnancy complications and parental and neonatal outcomes might differ, but perinatal morbidities will always increase. In addition, in twins, the differences in perinatal and neonatal outcomes between IVF pregnancies and natural pregnancies mostly narrowed or disappeared, which may indicate that pregnancy outcomes are greatly affected by multiple pregnancies, regardless of whether they are IVF or natural pregnancies. This finding may also be the result of the small number of cases.

### Limitation and strength of the study

The major strength of our study is the assessment of different infertility etiology on maternal characteristics and pregnancy outcomes in China. China has abolished the “one child” policy, and, since 2016, it has entered into the two-child policy era. As a result, the number of infants is expected to increase greatly, which may promote the demand for IVF [46]. Our findings have extremely important clinical implications and may provide guidance for couples and obstetricians in determining whether IVF is useful as a first-line treatment or as a last resort. Moreover, these findings may help in identifying likely perinatal and neonatal complications relating to different infertility etiologies and provide information for the underlying pathogenic mechanisms.

However, there are a few limitations of this study. First, the numbers of stillbirths and neonatal deaths were few; hence, these figures were not included in the main analysis, which may have given rise to the possibility of residual confounding in our results. Therefore, we could not accurately determine the severity of the effects of different infertility diagnoses on neonatal outcomes, nor could we identify the high-risk factors related to the long-term prognosis of the newborn. Another gap in the data that were available was the severity and treatment process of infertility. For example, data on the stage of endometriosis, baseline endocrine level, body mass index, duration of infertility, and ovarian stimulation protocol were incomplete. Although the IVF-ET method in our research was only frozen-embryo transfer, the data include both blastocyst and cleavage-stage embryo transfer. Additionally, each number of blastocysts and cleavage-stage embryos transferred was unknown, which would introduce bias in our results. Additionally, since the perinatal period starts at 28 complete weeks in China, the records of infants born before 28 weeks of gestational age are relatively incomplete. Therefore, only gestational ages above 28 weeks are included in this research. As a result, the exclusion of preterm birth between 24 and 28 weeks may lead to a reduction in the prevalence of PTB. In addition, we excluded unknown infertility diagnosis in the methods. Because of the substantial number of excluded cycles, those excluded due to unknown cause of infertility may introduce bias in the results. Moreover, we lack specific BMI data and have only data on prepregnancy obesity (BMI ≥28 kg/m^2^), which means higher BMI. Therefore, we could not add BMI as a continuous variable in the study groups. Finally, some information about environmental exposure (educational level, income level) was not included in this study, which may lead to bias. Further studies, particularly systematic reviews of observational studies such as the current study and prospective studies with adjustments for important confounders, will be required to confirm these initial findings. We are aware that some subgroups presents a limited sample size and likely limited statistical power.

## Conclusions

Taken together, through this retrospective, hospital-based cohort study, we verified that infertility etiology within the IVF population can affect maternal and neonatal outcomes among all births. Infertility etiology appears an additional risk factor for abnormal pregnancy outcomes besides the use of IVF techniques. Compared with natural pregnancies, IVF pregnancies with ovulation disorders have higher risks of adverse pregnancy and perinatal outcomes, as the rates of GDM, preeclampsia, postpartum hemorrhage, pPROM, and preterm birth were significantly increased, while IVF pregnancies with male infertility have lower risks of adverse pregnancy and poor perinatal outcomes. Doctors should fully inform patients of possible adverse pregnancy outcomes before they receive IVF. In addition, obstetricians should not only be aware of the increased risk of adverse outcomes with IVF but also pay attention to the specific complications related to the cause of infertility and provide timely treatment. These findings need to be considered with caution given the small sample size in some subgroups (particularly endometriosis and mixed infertility). Further studies, including prospective studies, are needed to confirm the role of the underlying infertility etiology and the severity of infertility in the increase in adverse outcomes with IVF after adjusting for important confounders.

## Supplementary Information

**Additional file 1: Supplemental Table 1**. Pregnancy outcomes among different infertility etiologies for IVF compared to spontaneous pregnancies in twins. **Supplemental Table 2**. Neonatal outcomes among different infertility etiologies for IVF compared to spontaneous pregnancies in Twins. **Supplemental Table 3**. cORs, aORs and 95% CIs of pregnancy outcomes among different infertility etiologies for IVF compared to spontaneous pregnancies in Twins. **Supplemental Table 4**. cORs, aORs and 95% CIs of neonatal outcomes among different infertility etiologies for IVF compared to spontaneous pregnancies in Twins

## Data Availability

The datasets used and/or analyzed during the current study are available from the corresponding author on reasonable request.

## Abbreviations

ART: Assisted reproductive technology
IVF: In vitro fertilization
BMI: Body mass index
GDM: Gestational diabetes mellitus
ICP: Intrahepatic cholestasis of pregnancy
pPROM: Preterm premature rupture of membranes
PB or PTB: Preterm birth
LBW: Low birthweight
SGA: Small for gestational age
NICU: Neonatal intensive care unit
CI: Confidence interval
aOR: Adjusted odds ratio
cOR: Crude odds ratio
NC: Not calculated due to low numbers
