# Supplementary material for: Pregnancy outcomes of Chinese women undergoing IVF with embryonic cryopreservation as compared to natural conception
Source: BMC Pregnancy Childbirth. 2021 Jan 9;21:39. doi: 10.1186/s12884-020-03486-7 (PMC7796545; doi:10.1186/s12884-020-03486-7)
Supplement: Supplementary file 1 — Additional file 1: Supplemental Table 1. Pregnancy outcomes among different infertility etiologies for IVF compared to spontaneous pregnancies in twins. Supplemental Table 2. Neonatal outcomes among different infertility etiologies for IVF compared to spontaneous pregnancies in Twins. Supplemental Table 3. cORs, aORs and 95% CIs of pregnancy outcomes among different infertility etiologies for IVF compared to spontaneous pregnancies in Twins. Supplemental Table 4. cORs, aORs and 95% CIs of neonatal outcomes among different infertility etiologies for IVF compared to spontaneous pregnancies in Twins [file 12884_2020_3486_MOESM1_ESM.docx]

|  | Control | | Ovulation disorder | | Tubal disease | | Endometriosis | | Male infertility | | Mixed infertility | |
| --- | --- | --- | --- | --- | --- | --- | --- | --- | --- | --- | --- | --- |
| Twins | N | % | N | % | N | % | N | % | N | % | N | % |
| Maternal outcomes | 98 |  | 133 |  | 261 |  | 33 |  | 136 |  | 39 |  |
| Gestational hypertension | 5 | 5.1 | 12 | 9 | 17 | 6.5 | 1 | 3 | 10 | 7.4 | 2 | 5.1 |
| Preeclampsia | 10 | 10.2 | 19 | 14.3 | 27 | 10.3 | 2 | 6.1 | 16 | 11.8 | 5 | 12.8 |
| Mild | 2 | 2 | 4 | 3 | 6 | 2.3 | 1 | 3 | 2 | 1.5 | 1 | 2.6 |
| Severe | 8 | 8.2 | 15 | 11.3 | 21 | 8 | 1 | 3 | 14 | 10.3 | 4 | 10.3 |
| Preterm eclampsia | 7 | 7.1 | 8 | 3 | 19 | 7.3 | 1 | 3 | 12 | 8.8 | 2 | 5.1 |
| GDM | 15 | 15.3 | 51 | 38.3* | 45 | 17.2 | 8 | 24.2 | 30 | 22.1 | 12 | 30.8^ |
| ICP | 3 | 3.1 | 4 | 3 | 8 | 3.1 | 0 | 0 | 4 | 2.9 | 1 | 2.6 |
| Placenta previa | 2 | 2 | 1 | 0.8 | 5 | 1.9 | 2 | 6.1 | 1 | 0.7 | 0 | 0 |
| Placental abruption | 3 | 3.1 | 1 | 0.8 | 1 | 0.4 | 0 | 0 | 1 | 0.7 | 0 | 0 |
| pPROM | 13 | 13.3 | 24 | 18.0 | 30 | 11.5 | 2 | 6.1 | 21 | 15.4 | 3 | 7.7 |
| Placenta accreta | 0 | 0 | 4 | 3 | 4 | 1.5 | 0 | 0 | 2 | 1.5 | 1 | 2.6 |
| Postpartum haemorrhage | 23 | 23.5 | 19 | 14.3 | 40 | 15.3 | 7 | 21.2 | 24 | 17.6 | 3 | 7.7^ |
| Polyhydramnios | 6 | 6.1 | 3 | 2.3^ | 2 | 0.8^ | 0 | 0 | 3 | 2.2 | 1 | 2.6 |
| Oligohydramnios | 1 | 1 | 2 | 1.5 | 1 | 0.4 | 0 | 0 | 1 | 0.7 | 0 | 0 |
| Chorioamnionitis | 1 | 1 | 3 | 2.3 | 1 | 0.4 | 0 | 0 | 0 | 0 | 0 | 0 |

Supplemental Table 1. Pregnancy outcomes among different infertility etiologies for IVF compared to spontaneous pregnancies in twins.

Note: GDM= Gestational diabetes mellitus; ICP=intrahepatic cholestasis of pregnancy; pPROM=preterm premature rupture of membranes; Mixed infertility refers to multiple infertility-related diagnosis. Data are presented as n (%) for dichotomous variable, *P* values were assessed by using the Pearson’s chi-square test, or Fisher’s exact test between each infertility subgroup vs controls ; *P*≤0.001=*; P<0.05=^. Nonsignificant numbers have not symbol.

Supplemental Table 2. Neonatal outcomes among different infertility etiologies for IVF compared to spontaneous pregnancies in Twins.

|  | Control | | Ovulation disorder | | Tubal disease | | Endometriosis | | Male infertility | | Mixed infertility | |
| --- | --- | --- | --- | --- | --- | --- | --- | --- | --- | --- | --- | --- |
| Twins | N | % | N | % | N | % | N | % | N | % | N | % |
|  | 98 |  | 133 |  | 161 |  | 33 |  | 136 |  | 39 |  |
| Gestational weeks | 35.6±2.4 |  | 35.8±2 |  | 36.2±1.8^ |  | 36.2±2.1 |  | 35.9±1.9 |  | 36.1±1.9 |  |
| Preterm birth | 54 | 55.1 | 70 | 52.6 | 99 | 37.9^ | 11 | 33.3^ | 64 | 47.1 | 16 | 41 |
| 34≤PTD<37 wk | 39 | 39.8 | 54 | 40.6 | 78 | 29.9 | 9 | 27.3 | 48 | 35.3 | 12 | 30.8 |
| 28≤PTD<34 wk | 15 | 15.3 | 16 | 12 | 20 | 7.7^ | 2 | 6.1 | 16 | 11.8 | 4 | 10.3 |
| Birthweight, g | 2413.5±464.6 |  | 2537±486^ |  | 2580±419* |  | 2557±453 |  | 2514±447 |  | 2494±449 |  |
| <2500 g | 61 | 62.2 | 71 | 53.4 | 132 | 50.6^ | 17 | 51.5 | 73 | 53.7 | 22 | 56.4 |
| <1500 g | 10 | 10.2 | 8 | 6 | 13 | 5 | 2 | 6.1 | 9 | 6.6 | 3 | 7.7 |
| SGA | 33 | 33.7 | 36 | 27.1 | 80 | 30.7 | 10 | 30.3 | 35 | 25.7 | 14 | 35.9 |
| Macrosomia | 0 | 0 | 0 | 0 | 0 | 0 | 0 | 0 | 0 | 0 | 0 | 0 |
| Body length, cm | 45.82±3.43 |  | 46.70±3.05^ |  | 46.96±2.56^ |  | 46.65±3.00 |  | 46.51±2.95 |  | 46.41±3.50 |  |
| 1-Minute Apgar score≤7 | 2 | 2 | 5 | 3.8 | 4 | 1.5 | 2 | 6.1 | 6 | 4.4 | 2 | 5.1 |
| 5-Minute Apgar score≤7 | 0 | 0 | 2 | 1.5 | 1 | 0.4 | 0 | 0 | 3 | 2.2 | 1 | 2.6 |
| NICU admission | 53 | 54.1 | 78 | 58.6 | 139 | 53.3 | 15 | 45.5 | 78 | 57.4 | 20 | 51.3 |

Note: PTD=preterm delivery; SGA=small for gestational age( birthweight below the 10th percentile for gestational age); Macrosomia=birth weight≥4000g;

Mixed infertility refers to multiple infertility-related diagnosis.

Data are presented as means±SDs for continuous variables and n (%) for dichotomous variables. *P* values were assessed by using the Pearson’s chi-square test, Fisher’s exact test or the *t* test between each infertility subgroup vs controls ; *P*≤0.001=*; P<0.05=^. Nonsignificant numbers have not symbol.

Supplemental Table 3. cORs, aORs and 95% CIs of pregnancy outcomes among different infertility etiologies for IVF compared to spontaneous pregnancies in Twins.

|  | Ovulation disorder | | | | Tubal disease | | | | Endometriosis | | | | Male infertility | | | | Mixed infertility | | | | |
| --- | --- | --- | --- | --- | --- | --- | --- | --- | --- | --- | --- | --- | --- | --- | --- | --- | --- | --- | --- | --- | --- |
| Twins | cOR | aOR | 95% CI | | cOR | aOR | 95% CI | | cOR | aOR | 95% CI | | cOR | aOR | 95% CI | | cOR | aOR | 95% CI | | |
| GH | **0.42** | 3.36 | 0.96 | 11.82 | **0.60** | 2.38 | 0.69 | 8.16 | 1.33 | 0.99 | 0.10 | 9.92 | **0.52** | 2.67 | 0.72 | 9.96 | 0.77 | 2.02 | 0.31 | 12.95 |  |
| Preeclampsia | **0.18** | 0.94 | 0.37 | 2.38 | **0.26** | 0.64 | 0.26 | 1.55 | 0.47 | 0.37 | 0.07 | 1.90 | **0.23** | 0.78 | 0.30 | 2.02 | **0.21** | 0.80 | 0.23 | 2.80 |  |
| Mild | **0.31** | 0.74 | 0.11 | 4.89 | **0.40** | 0.56 | 0.09 | 3.28 | 0.30 | 0.73 | 0.06 | 9.65 | 0.63 | 0.33 | 0.03 | 2.75 | 0.36 | 0.55 | 0.04 | 7.11 |  |
| Severe | **0.16** | 1.07 | 0.38 | 3.07 | **0.23** | 0.71 | 0.26 | 1.95 | 0.65 | 0.27 | 0.03 | 2.41 | **0.18** | 1.04 | 0.37 | 3.01 | **0.18** | 0.99 | 0.24 | 4.00 |  |
| PPE | **0.05** | 1.38 | 0.48 | 3.95 | **0.10** | 0.68 | 0.24 | 1.93 | 0.25 | 0.28 | 0.03 | 2.54 | **0.08** | 0.88 | 0.29 | 2.67 | **0.14** | 0.45 | 0.08 | 2.52 |  |
| GDM | **0.32** | **3.74** | **1.73** | **8.09** | 0.95 | 1.23 | 0.58 | 2.63 | 0.62 | 1.61 | 0.55 | 4.72 | 0.70 | 1.62 | 0.73 | 3.61 | **0.45** | **2.77** | **1.03** | **7.48** |  |
| ICP | **0.09** | 0.54 | 0.11 | 2.80 | **0.09** | 0.54 | 0.12 | 2.41 | NC | NC | NC | NC | **0.09** | 0.48 | 0.09 | 2.50 | **0.10** | 0.43 | 0.04 | 4.77 |  |
| Placenta previa | 1.85 | 0.23 | 0.02 | 3.39 | 0.72 | 0.55 | 0.08 | 3.90 | **0.22** | 1.65 | 0.17 | 16.23 | 1.90 | 0.21 | 0.01 | 2.93 | NC | NC | NC | NC |  |
| Placental abruption | 1.84 | 0.12 | 0.01 | 1.51 | 3.61 | **0.08** | **0.01** | **0.87** | NC | NC | NC | NC | 0.26 | 0.12 | 0.01 | 1.39 | NC | NC | NC | NC |  |
| pPROM | **0.11** | 2.13 | 0.88 | 5.13 | **0.19** | 1.26 | 0.54 | 2.97 | 0.09 | 0.69 | 0.13 | 3.52 | **0.14** | 1.80 | 0.73 | 4.48 | **0.30** | 0.90 | 0.22 | 3.79 |  |
| Placenta accreta | NC | NC | NC | NC | NC | NC | NC | NC | NC | NC | NC | NC | NC | NC | NC | NC | NC | NC | NC | NC |  |
| PH | **0.57** | 1.45 | 0.63 | 3.35 | **0.52** | 1.76 | 0.82 | 3.80 | **0.35** | 2.78 | 0.91 | 8.47 | **0.44** | 2.10 | 0.91 | 4.88 | 1.13 | 1.02 | 0.25 | 4.13 |  |
| Polyhydramnios | 0.42 | 0.43 | 0.08 | 2.23 | 1.26 | 0.14 | 0.02 | 0.89 | NC | NC | NC | NC | 0.43 | 0.38 | 0.07 | 2.06 | 0.37 | 0.49 | 0.05 | 5.33 |  |
| Oligohydramnios | 2.43 | 0.33 | 0.03 | 4.22 | **9.65** | 0.09 | 0.01 | 1.52 | NC | NC | NC | NC | 5.01 | 0.18 | 0.01 | 3.26 | NC | NC | NC | NC |  |
| Chorioamnionitis | 0.58 | 1.39 | 0.08 | 22.88 | 3.50 | 0.26 | 0.01 | 6.51 | NC | NC | NC | NC | NC | NC | NC | NC | NC | NC | NC | NC |  |

Note: *Logistic regression analysis was adjusted for age, gravidity, parity, pre-pregnancy obesity, birth plurality, and history of previous caesarean section. CI=confidence interval; cOR=crude odds ratio; aOR=adjusted odds ratio. Mixed infertility refers to multiple infertility-related diagnosis; GH=gestational hypertension; PPE=preterm preeclampsia; GDM=gestational diabetes mellitus; pPROM=preterm premature rupture of membranes; PH=postpartum haemorrhage; ICP=intrahepatic cholestasis of pregnancy; The reference group of logistic regression is spontaneous control pregnancy.

Bold indicates significant differences; NC=not calculated due to low numbers.

Supplemental Table 4. cORs, aORs and 95% CIs of neonatal outcomes among different infertility etiologies for IVF compared to spontaneous pregnancies in Twins.

|  | Ovulation disorder | | | | Tubal disease | | | | Endometriosis | | | | Male infertility | | | | Mixed infertility | | | |
| --- | --- | --- | --- | --- | --- | --- | --- | --- | --- | --- | --- | --- | --- | --- | --- | --- | --- | --- | --- | --- |
| Twins | cOR | aOR | 95% CI | | cOR | aOR | 95% CI | | cOR | aOR | 95% CI | | cOR | aOR | 95% CI | | cOR | aOR | 95% CI | |
| Preterm birth | **0.06** | 1.33 | 0.72 | 2.47 | **0.10** | 0.74 | 0.42 | 1.31 | **0.13** | 0.66 | 0.27 | 1.63 | **0.07** | 1.14 | 0.61 | 2.12 | **0.09** | 0.89 | 0.38 | 2.06 |
| 34≤PTD<37 wk | **0.07** | 1.01 | 0.54 | 1.89 | **0.11** | 0.63 | 0.35 | 1.14 | **0.12** | 0.58 | 0.23 | 1.47 | **0.09** | 0.83 | 0.44 | 1.57 | **0.10** | 0.65 | 0.27 | 1.57 |
| 28≤PTD<34 wk | **0.11** | 2.05 | 0.76 | 5.51 | **0.18** | 1.26 | 0.49 | 3.24 | **0.23** | 1.23 | 0.23 | 6.65 | **0.11** | 2.38 | 0.86 | 6.57 | **0.13** | 2.24 | 0.56 | 8.94 |
| Birthweight <2500 g | **0.04** | 0.86 | 0.47 | 1.60 | **0.04** | 0.76 | 0.43 | 1.36 | **0.04** | 0.86 | 0.36 | 2.05 | **0.04** | 0.89 | 0.48 | 1.66 | **0.04** | 1.01 | 0.44 | 2.34 |
| Birthweight <1500 g | **0.11** | 1.03 | 0.31 | 3.42 | **0.13** | 0.84 | 0.28 | 2.53 | **0.11** | 1.32 | 0.23 | 7.62 | **0.10** | 1.32 | 0.40 | 4.40 | **0.08** | 1.56 | 0.32 | 7.59 |
| SGA | **0.13** | 0.62 | 0.32 | 1.20 | **0.11** | 0.72 | 0.39 | 1.32 | **0.11** | 0.74 | 0.29 | 1.88 | **0.14** | 0.57 | 0.29 | 1.11 | **0.09** | 0.90 | 0.38 | 2.15 |
| 1-Minute Apgar score≤7 | **0.25** | 6.88 | 0.81 | 58.29 | 0.62 | 3.07 | 0.35 | 26.69 | **0.15** | **20.50** | **1.66** | **253.72** | **0.21** | **10.61** | **1.21** | **93.25** | **0.18** | **18.78** | **1.45** | **242.89** |
| NICU admission | **0.09** | 1.66 | 0.90 | 3.09 | **0.11** | 1.34 | 0.75 | 2.37 | **0.15** | 1.07 | 0.45 | 2.55 | **0.09** | 1.62 | 0.87 | 3.03 | **0.12** | 1.33 | 0.58 | 3.05 |

Note: *Logistic regression analysis was adjusted for age, gravidity, parity, pre-pregnancy obesity, birth plurality, and history of previous caesarean section. CI=confidence interval; aOR=adjusted odds ratio.

Mixed infertility refers to multiple infertility-related diagnosis; PTD=preterm delivery; SGA=small for gestational age( birthweight below the 10th percentile for gestational age); NICU= neonatal intensive care unit；Macrosomia=birth weight≥4000g；The reference group of logistic regression is spontaneous control pregnancy. Bold indicates significant differences; NC=not calculated due to low numbers.
